# Endothelial differentiation of bone marrow mesenchyme stem cells applicable to hypoxia and increased migration through Akt and NFκB signals

**DOI:** 10.1186/s13287-017-0470-0

**Published:** 2017-02-07

**Authors:** Cheng Liu, An-Ly Tsai, Ping-Chia Li, Chia-Wei Huang, Chia-Ching Wu

**Affiliations:** 1 Hyperbaric Oxygen Therapy Center, Chi-Mei Medical Center, Tainan, Taiwan; 2 Division of Plastic Surgery, Chi-Mei Medical Center, Tainan, Taiwan; 3 Department of Electrical Engineering, Southern Taiwan University of Science and Technology, Tainan, Taiwan; 4 Department of Cell Biology and Anatomy, College of Medicine, National Cheng Kung University, Tainan, Taiwan; 5 Department of Occupational Therapy, I-Shou University, Kaohsiung, Taiwan; 6 School of Medicine for International Students, I-Shou University, Kaohsiung, Taiwan; 7 Institute of Basic Medical Sciences, College of Medicine, National Cheng Kung University, Tainan, Taiwan; 8 Medical Device Innovation Center, National Cheng Kung University, Tainan, Taiwan

**Keywords:** Stem cell, Endothelial differentiation, Hypoxia, Migration, Akt, Nuclear factor-κB

## Abstract

**Background:**

Bone marrow mesenchymal stem cells (MSCs) and endothelial progenitor cells (EPCs) are used to repair hypoxic or ischemic tissue. However, the underlining mechanism of resistance in the hypoxic microenvironment and the efficacy of migration to the injured tissue are still unknown. The current study aims to understand the hypoxia resistance and migration ability of MSCs during differentiation toward endothelial lineages by biochemical and mechanical stimuli.

**Method:**

MSCs were harvested from the bone marrow of 6–8-week-old Sprague–Dawley rats. The endothelial growth medium (EGM) was added to MSCs for 3 days to initiate endothelial differentiation. Laminar shear stress was used as the fluid mechanical stimulation.

**Results:**

Application of EGM facilitated the early endothelial lineage cells (eELCs) to express EPC markers. When treating the hypoxic mimetic desferrioxamine, both MSCs and eELCs showed resistance to hypoxia as compared with the occurrence of apoptosis in rat fibroblasts. The eELCs under hypoxia increased the wound closure and C-X-C chemokine receptor type 4 (CXCR4) gene expression. Although the shear stress promoted eELC maturation and aligned cells parallel to the flow direction, their migration ability was not superior to that of eELCs either under normoxia or hypoxia. The eELCs showed higher protein expressions of CXCR4, phosphorylated Akt (pAkt), and endogenous NFκB and IκBα than MSCs under both normoxia and hypoxia conditions. The potential migratory signals were discovered by inhibiting either Akt or NFκB using specific inhibitors and revealed decreases of wound closure and transmigration ability in eELCs.

**Conclusion:**

The Akt and NFκB pathways are important to regulate the early endothelial differentiation and its migratory ability under a hypoxic microenvironment.

**Electronic supplementary material:**

The online version of this article (doi:10.1186/s13287-017-0470-0) contains supplementary material, which is available to authorized users.

## Background

Hypoxic or ischemic injury causes cell death by oxidative stress and cellular signals to trigger tissue necrosis and subsequently life-long dysfunctions [1–5]. The injured tissues produce cytokines and chemokines to recruit stem or progenitor cells for repairing the damaged sites. However, the number of endogenous therapeutic cells is usually not sufficient to recover a large injury site. Stem or progenitor cells have been applied to rescue ischemic injury in clinical trials, such as myocardial infarction or stroke [6, 7]. Bone marrow mesenchymal stem cells (MSCs) have the characteristics of self-renewal and multipotency [8–10]. MSCs are potent therapeutic sources to differentiate or transdifferentiate into other therapeutic lineages for neovascular genesis of new vessels to repair damaged tissues [11–13].

Cell apoptosis under hypoxia is regulated by mitogen-activated protein kinase (MAPK), nuclear factor-κB (NFκB), phosphatidylinositol 3-kinase (PI3K)/Akt, or the release of cytochrome C to activate the apoptotic cascades [14, 15]. Under hypoxia, reactive oxygen species are produced to degrade the NFκB inhibitor (IκB) into RelA/p50 dimer for nuclear translocation. Hypoxia also regulates hypoxia inducible factor-1 (HIF-1) activity via PI3K/Akt or MAPK signaling in an oxygen-independent manner. The interaction and crosstalk of HIF-1 and NFκB signals are important for immune responses, inflammation, and anti-apoptosis [16].

In hypoxic tissue, stromal cell-derived factor-1 (SDF-1) and C-X-C chemokine receptor type 4 (CXCR4) are important factors for cell migration. The damaged tissues secrete SDF-1 to attract CXCR4-expressed cells, particularly the therapeutic progenitors [17]. Conversely, MSCs originate from the bone marrow microenvironmental niche with exhibiting of low oxygen tension [18, 19]. In-vitro culture of MSCs under hypoxic conditions (low oxygen tension) showed benefits in maintaining cell self-renewal, migration, vascular tube formation, and release of paracrine factors for chemotactic and proangiogenic properties [20–22]. Endothelial progenitor cells (EPCs) are classified into early EPCs and late EPCs which can be isolated from peripheral blood or bone marrow [23, 24]. Upon tissue damage, EPCs are mobilized from the bone marrow and migrated into the hypoxic region to regenerate the vascular structure and restore tissue function [25, 26]. EPCs participate in the reendothelialization of angiogenesis as well as of vasculogenesis by differentiating into mature endothelial cells (ECs) [27–29]. For the injected EPCs, recruitment and incorporation into the ischemic region is essential for achieving the beneficial outcomes [30, 31]. However, the migration and functional roles of therapeutic cells in MSCs as well as in different stages of EPCs under a hypoxic microenvironment are still not clear.

The combination of biochemical and mechanical stimuli promotes several adult stem cells, including placenta-derived multipotent cells (PDMCs) [32] and adipose-derived stem cells (ASCs) [33, 34], to switch their MSC characteristics toward endothelial lineage cells (ELCs). ELCs are defined as mixture cells for cell transplantation without sorting of different endothelial populations after endothelial differentiation [33]. The application of endothelial growth medium (EGM) to these adult stem cells induces expression of early EPC markers named early ELCs (eELCs). After a subsequent mechanical stimulation of laminar shear stress (LSS), ELCs showed mature EC characteristics of forming vascular tube-like stucture and uptake of lipoproteins [32]. However, the differentiation of MSCs using this approach and their characteristics under hypoxia are still unknown. Desferrioxamine (DFO), an iron chelator, is known to upregulate hypoxia signals by stabilizing the HIF-1 activity [35] and to reduce free radical-mediated cell injury [36]. In the current study, we are interested to know the anti-apoptosis and migration abilities of MSCs and their differentiated ELCs under hypoxic microenvironments. We hypothesize that MSCs and their ELCs can resist hypoxia and able to migrate toward the injury site via a specific signaling pathway for repairing the damaged tissue. The understanding of cellular responses and potential signals for hypoxia in MSCs and ELCs may benefit the clinical preconditioning of these therapeutic cells for better repair outcomes.

## Methods

### Cell culture and differentiation

Bone marrow-derived MSCs were harvested from femoral bone marrow of 8-week-old Sprague–Dawley (SD) rats. Briefly, the cells were flushed from the femoral bone and collected into Dulbecco’s modified Eagle medium (DMEM; Invitrogen) supplemented with 10% fetal bovine serum (FBS; Hyclone) and 1% penicillin–streptomycin (Invitrogen), and seeded on a 100-mm Petri dish. The detached cells were removed after culture for 24 hr. The adhered cells were characterized and defined as rat MSCs after confirmation of stem cell markers and differentiation ability [32]. The MSCs were used between passages 2 and 5 in the current study. The NRK49F fibroblast cell line (ATCC) was cultured in DMEM supplement with 10% FBS and 1% penicillin–streptomycin to represent the rat stromal cells. To induce endothelial differentiation, eELCs were induced by culturing MSCs in medium 199 (M199; Invitrogen) supplemented with 20% FBS, EGM (Lonza), and 1% penicillin–streptomycin under a static condition for 3 days [32, 34]. The maturation of ELCs was induced by subjecting the eELCs to LSS (12 dyn/cm^2^) for 24 hr using the flow chamber system [32].

### Cell treatments under in-vitro hypoxic microenvironments

The hypoxic mimetic DFO (Sigma-Aldrich) was used to create an in-vitro hypoxic microenvironment with different dosages (10, 20, 50 μM) [37]. The fibroblasts, MSCs, and differentiated cells were rinsed with phosphate-buffered saline (PBS) and then exchanged to fresh DMEM containing 1% FBS and different dosages of DFO. The hypoxic microenvironment was also created by placing cells in a hypoxia incubator (Autoflow 4950; NuAire Inc.) and reducing the oxygen concentration to 2%. Low oxygen tension hypoxia was created by mixing 5% CO_2_ and replacing oxygen with N_2_ in the hypoxia incubator. Upon blockage of potential signaling pathways using specific inhibitors, the CXCR4 signal was inhibited by CXCR4 antagonist AMD3100 (Sigma-Aldrich). The PI3K/Akt inhibitor LY294006 (10 μM; Sigma-Aldrich) and the antioxidant pyrrolidinedithiocarbamate (PDTC, 10 μM; Sigma-Aldrich) were used to inhibit Akt phosphorylation and NFκB activity, respectively. To abolish the stimulation upon DFO application, cells were pretreated with specific inhibitors (LY294006, PDTC, or AMD3100) for 30 min, and then DFO applied for the indicated time.

### Flow cytometry assessments

Flow cytometry was used to quantify the cell apoptosis and CD surface markers. The early stage of cell apoptosis was further confirmed using flow cytometry and by positive staining with annexin V and negative staining with propidium iodide (PI) [38]. The fibroblasts, MSCs, and eELCs treated with DFO were resuspended and incubated with fluorescein isothiocyanate (FITC)-labeled annexin V antibody and PI (Strong Biotech Corporation) in the dark at 4 °C for 15 min. The labeled cells were measured by flow cytometry (FACScan; BD Biosciences) and analyzed by WinMDI software. Percentages of cells with positive staining for annexin V and negative staining for PI were calculated to identify the apoptotic cells.

To quantify the MSC characteristics, the specific antibodies against CD34 (1:40; Abcam), CD45 (1:100; BD Pharmingen), and CD90 (1:50; BD Pharmingen) were measured in MSCs, eELCs, and fibroblasts. Antibodies for Flt (VEGFR1, 1:100; Abcam) and Flk (VEGFR2, 1:100; Abcam) were used as the positive markers for early EPCs, whereas PECAM-1 (CD31, 1:40; BD Pharmingen) was labeled for the late EPCs or mature ECs. In brief, the trypsinized cells were incubated with specific antibodies in the dark at 4 °C for 30 min and then rinsed with wash buffer (PBS with 0.2% BSA) by short centrifuge. The fluorescent intensities of labeled cells were quantified by flow cytometry (FACS Calibur; BD Biosciences), counting 10,000 cells in each sample. The NRK49F fibroblast was defined as the negative stained threshold to distinguish the positive cells in MSCs and eELCs.

### Measurement of gene and protein expressions

The reverse transcription polymerized chain reaction (RT-PCR) and quantitative real-time PCR (qPCR) were performed to measure the gene (mRNA) expressions according to a previous study [32]. Briefly, the cells were lysed by Trizol (Invitrogen) to isolate the mRNA and then reverse transcripted into cDNA using Super Script III (Invitrogen). The specific gene expressions were amplified and detected by the Taq-PCR (GeneDirex) system with specific primers [33]. For qPCR, the SYBR™ green master mix (Thermo Fisher) was used to amplify the specific genes with forward (F) and reverse (R) primer sequences for *Flt* (F: GAAGAGTGGGTCGTCATTCC, R: GTAGCC ATGCACCGAATAGC), *Flk* (F: CGGGAAACTACACGGTCATC, R: GGGAGGGTT GGCATAGACT), *von Willebrand factor (vWF)* (F: CAGGGCTCTACCAGGATGAA, R: TTTGCTGCGGTG AGACAA), and *GAPDH* (F: TGCCACTCAGAAGACTGTGG, R: ACGGATACATTG GGGGTAGG). The relative gene expressions were calculated using the 2^–ΔΔCt^ method normalized to the housekeeping gene GAPDH. The endothelial differentiation was further confirmed by the expression levels of early EPC markers for *Flt* and *Flk*. *vWF* and *PECAM-1* were used to indicate the gene expression of mature EC markers.

The protein expressions for intracellular signaling were assessed by western blotting. The cells were rinsed twice with cold PBS and then lysed with RIPA buffer containing protease inhibitors. Cell lysates were analyzed by sodium dodecyl sulfate polyacrylamide gel electrophoresis (SDS-PAGE) with 10% cross-linking gel, and then transferred into nitrocellulose membranes (Bio-Rad). The membranes were blocked by 5% dry milk in TBS with 0.5% Tween 20 for 90 min. For specific protein detection, membranes were hybridized with specific primary antibodies overnight at 4 °C. Bound primary antibodies were detected using appropriate secondary antibodies coupled to horseradish peroxidase (Sigma-Aldrich) and by an ECL detection system (Millipore). The antibody against poly-ADP ribose polymerase (PARP, 1:1000; Cell Signaling), a downstream protein which is cleaved in apoptotic cell via caspase signals, was used to detect the cleaved PARP for indicating cell apoptosis. The expression of CXCR4 was assessed by specific CXCR4 antibody (1:1000; Abcam). The phosphorylation levels of Akt signal were detected by the antibody against the phospho-Akt (pAkt, 1:500; Cell Signaling) and normalized to total form Akt (tAkt, 1:100; Santa Cruz) protein. NFκB signaling was measured by NFκB p65 (1:500; Santa Cruz) and IκBα (1:500; Santa Cruz) antibody. The fold changes of cleaved PARP, NFκB p65, and IκBα were normalized to β-actin. The nuclear and cytoplasmic fractions were extracted using a nuclear and cytoplasmic extraction kit (G-Biosciences) to demonstrate the nuclear translocation of NFκB in accordance with the user instructions. Lamin A/C antibody (1:500; Santa Cruz) was used to indicate the successful isolation of nuclear protein in western blotting.

### Assessment of cell migration ability

The ability of stem cells to migrate into the lesion site is important for tissue protection and regeneration. We utilized wound closure and Boyden chamber assays to assess the migration of MSCs and ELCs. For the wound closure assay, the MSCs and differentiated ELCs were cultured on a six-well plate until full confluence and then created a “wound” by scratching a gap using a pipette tip. After rinsing with PBS, cells were then incubated in fresh DMEM with or without DFO for 24 hr. For treatment with inhibitors, the inhibitors were applied to the confluent cells for 30 min to create a wound for cells to close under normoxia or hypoxia conditions. The phase images for wounds were recorded at 0 and 24 hr by ImageJ software (Image J). The percentage of wound closure (%) was measured by quantifying wound areas at 24 hr (A_24_) and deductive to the initial time points (A_0_) using the equation (A_0_ – A_24_) / A_0_ [39].

The Boyden chamber (48-Well Micro Chemotaxis Chamber; Neuro Probe) was used to detect chemotaxis and transmigration in MSCs and endothelial differentiated cells. Cells were resuspended and counted for 4 × 10^5^ cells/ml to load into the upper compartment of the Boyden chamber. The migration ability was measured by counting the cells that migrated through 8-μm pore membranes (Neuro Probe) to the lower compartment after incubation for 6 hr with medium with or without 50 μM of DFO. Specific inhibitors were pretreated to the cells for 30 min before resuspending and loading into the Boyden chamber. The transmigration was quantified after dissembling the chamber, fixing cells with 4% paraformaldehyde for 5 min, and then staining with Giemsa for 15 min. Images were taken by microscope (CX31; Olympus) and quantified using ImageJ software (ImageJ) with normalizing to the transmigrated cell numbers under normoxia (without DFO).

### Statistical analyses

For all experiments, at least three independent groups were performed to demonstrate a consistent outcome. All data were expressed as the mean ± standard SEM. Statistical analysis was performed using one-way analysis of variance (ANOVA) and *p* < 0.05 was considered statistically significant using Origin statistical software (version 8.5; OriginLab).

## Results

### MSCs and early differentiated ELCs resistant to hypoxia than somatic cells

**Fig. 1:**
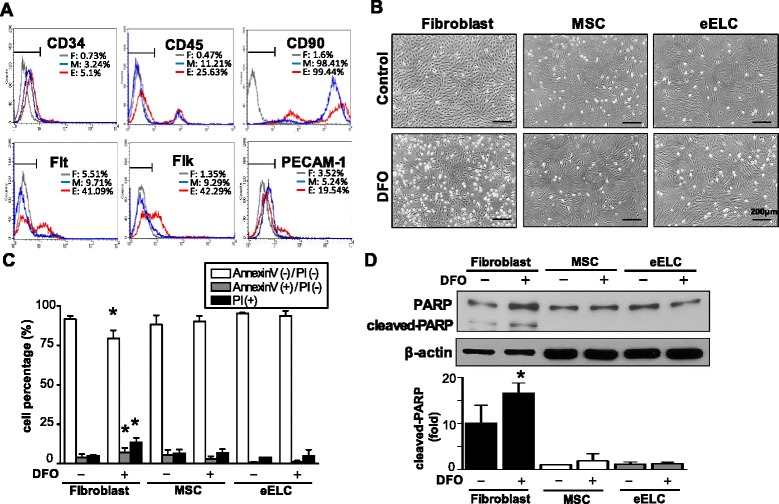
Surface CD markers were characterized by flow cytometry in fibroblasts (*F*), bone marrow mesenchymal stem cells (*M*), and early endothelial lineage cells (*E*) (**a**). Fibroblasts, but not mesenchymal stem cells (*MSC*) or early endothelial lineage cells (*eELC*), showed apoptotic morphology in phase images after treating with desferrioxamine (*DFO*) for 48 hr (**b**). The DFO-increased apoptotic (Annexin V(+)/PI(−)) and dead (PI(+)) somatic fibroblasts were quantified by flow cytometry (**c**) and confirmed by the protein expression of cleaved PARP (**d**). *Scale bar*: 200 μm. *Significant from the same cell under regular culture conditions, *p* < 0.05. *PARP* poly-ADP ribose polymerase, *PI* propidium iodide

The MSC surface CD markers were observed by positive staining of CD90 (98.41%) and negative staining of CD34, CD45, Flt, Flk, and CD31 in MSCs (Fig. 1a). The eELCs were differentiated by culturing the MSCs in EGM for 3 days. The early EPC markers were increased for both Flt (41.09%) and Flk (42.29%) in eELCs as compared with the expression patterns in MSCs. The eELCs also showed a partial increase of PECAM-1-positive cells (19.54%). Although the CD90 intensity slightly decreased in eELCs, the protein expression level was still showed positive as compared with the negative stained fibroblasts.

To understand the effect of hypoxia in MSCs and their derived cells, DFO (50 μM) was treated for 48 hr and significant cellular morphological changes were not observed in MSCs and eELCs as compared with the rat fibroblasts (Fig. 1b). However, membrane blebbing and shrinkage of the cell body were observed in fibroblasts, suggesting that DFO might cause cell damage or apoptosis in somatic cells. The annexin V/PI double staining flow cytometry was used to assess the cell apoptosis and death under DFO treatment (Fig. 1c). In regular culture condition (normoxia), the fibroblasts, MSCs, and eELCs have more than 90% living cells (annexin V/PI double-negative). The living fibroblasts were significantly decreased after hypoxia (DFO) for 48 hr and switched to the early apoptotic cells (annexin V-positive/PI-negative) and dead cells (PI-positive) (Fig. 1c). On the contrary, no significant difference of MSCs and eELCs occurred after treatment with DFO. Cleaved PARP was observed and confirmed the hypoxia-induced cell apoptosis in fibroblasts, but not in either MSCs or eELCs (Fig. 1d). These results suggest that MSCs are resisted to the hypoxic microenvironments and the early differentiation of ELCs also endures in DFO treatment.

### Hypoxic microenvironment enhanced migration of eELCs

**Fig. 2:**
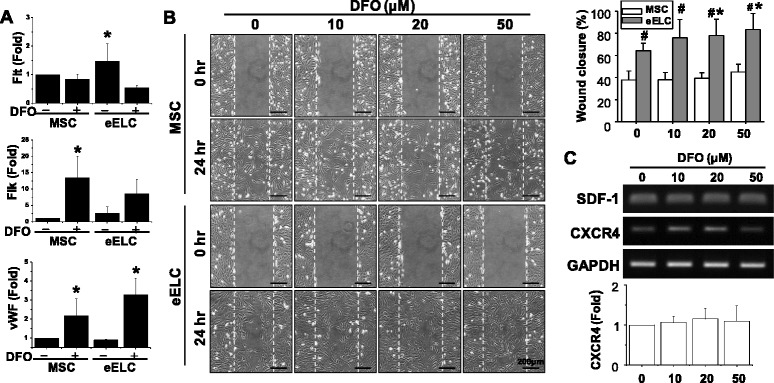
DFO treatment enhanced gene expression of *Flk* and *vWF* (**a**). eELCs showed higher wound closure ability (%) than MSCs in normoxia (DFO = 0), and hypoxia (DFO) further increased the closure performance in a dose-dependent manner (**b**). *CXCR4* gene expression was increased after treating eELCs with DFO for 24 hr (**c**). *Scale bar*: 200 μm. *Significant from the same cell under regular culture condition (without DFO), *p* < 0.05; #significant from MSCs under the same treatment, *p* < 0.05. *DFO* desferrioxamine, *MSC* mesenchymal stem cells, *eELC* early endothelial lineage cells, *vWF* von Willebrand factor, *SDF-1* stromal cell-derived factor-1, *CXCR4* C-X-C chemokine receptor type 4

Treatment of DFO for 24 hr in eELCs further enhanced endothelial gene expressions, especially in the mature EC marker vWF (Fig. 2a). The qPCR results showed that the increase of Flt gene expression in eELCs was inhibited after DFO treatment, but Flk gene expression was relatively increased in both MSCs and eELCs. We were further interested to know the beneficial effects of hypoxia in eELCs. Cell migration was measured by wound closure assay in both MSCs and eELCs with different dosages of DFO (0, 10, 20, 50 μM). The MSCs and eELCs showed similar proliferation potential as indicated by BrdU and Ki67 staining (Additional file 1: Figure S1). Under normoxia (without DFO), the eELCs already showed a better wound closure rate than MSCs at 24 hr after wounding (Fig. 2b). The MSCs showed about 37% of closure by migrating into the empty wound area, whereas the eELCs presented around 63% of wound closure migration. With DFO treatment at 20 and 50 μM, eELC migration to close the wound increased to about 80% and showed a dose-dependent manner. DFO treatment also increased minor cell migration in MSCs, but not as obvious as that in eELCs. Because SDF-1/CXCR4 chemotaxis plays a critical role in stem/progenitor cell migration, we further assessed the gene expression of SDF-1 and CXCR4 with different dosages of DFO. The chemoattractant SDF-1 level showed no difference, but its receptor CXCR4 was upregulated in responding to the DFO dosage (Fig. 2c).

### Shear stress promoted ELC maturation, but decreased cell migration ability

**Fig. 3:**
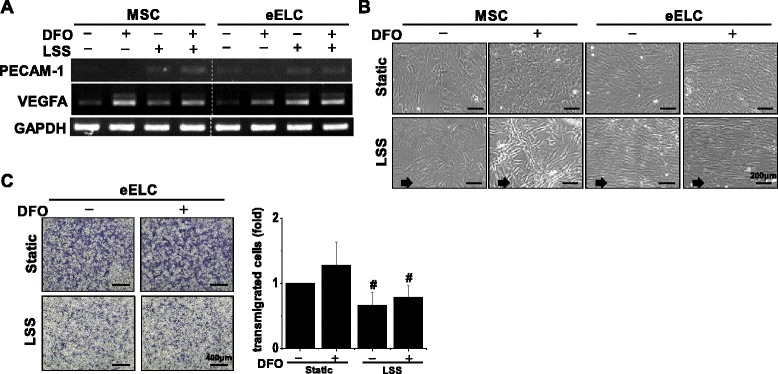
Mature endothelial characteristics were observed after the eELCs were subjected to laminar shear stress (*LSS*) for 24 hr. The *PECAM-1* gene (**a**) and cellular alignment in responding to the flow direction (**b**) were increased under LSS. However, the Boydon chamber assay demonstrated decreases of cell transmigration after LSS stimulation (**c**). *Scale bar*: (**b**) 200 μm; (**c**) 400 μm. #Significant from static eELC under hypoxia (DFO, 50 μM), *p* < 0.05. *DFO* desferrioxamine, *MSC* mesenchymal stem cells, *eELC* early endothelial lineage cells

Because shear stress may provide the synergistic effect in endothelial differentiation for other adult stem cells [32, 33], we assessed the interaction of DFO and LSS (12 dyn/cm^2^) in both MSCs and eELCs. Either DFO or LSS treatment increased the VEGFA mRNA expressions in MSCs, but their combination did not show further enhancement of VEGFA (Fig. 3a). VEGFA also increased in eELCs after treatment with DFO or LSS. On the contrary, the combination of DFO and LSS increased the mature EC marker, PECAM-1, in MSCs. LSS alone induced PECAM-1 expression which suggests that shear stress can promote the maturation of eELCs. After applying LSS for 24 hr, the phase-contrast cell images showed a parallel orientation of elongated eELCs to indicate their responses to the flow as mature endothelium (Fig. 3b). The occurrence of DFO during LSS stimulation did not alter eELC morphology. Although LSS promoted eELC maturation, these mature cells showed decreases of transmigration abilities (stained cells) regardless of DFO treatment in the Boyden chamber assay (Fig. 3c). These results suggest that mature ECs do not enhance their migration ability as compared with the early ELCs under either the normoxia or hypoxia condition.

### Involvement of Akt and NFκB signals in eELC migration

**Fig. 4:**
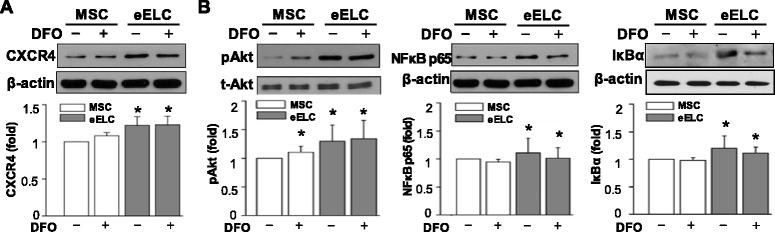
CXCR4 protein expression was increased in eELCs under both normoxia (without DFO) and hypoxia (with DFO) conditions (**a**). Phosphorylated Akt (pAkt) was increased in MSCs under DFO treatment (**b**). eELCs showed higher protein expressions of pAkt, NFκB p65, and IκBα than MSCs regardless of DFO treatment. *Significant from MSCs under regular culture condition, *p* < 0.05. *DFO* desferrioxamine, *MSC* mesenchymal stem cells, *eELC* early endothelial lineage cells, *CXCR4* C-X-C chemokine receptor type 4

**Fig. 5:**
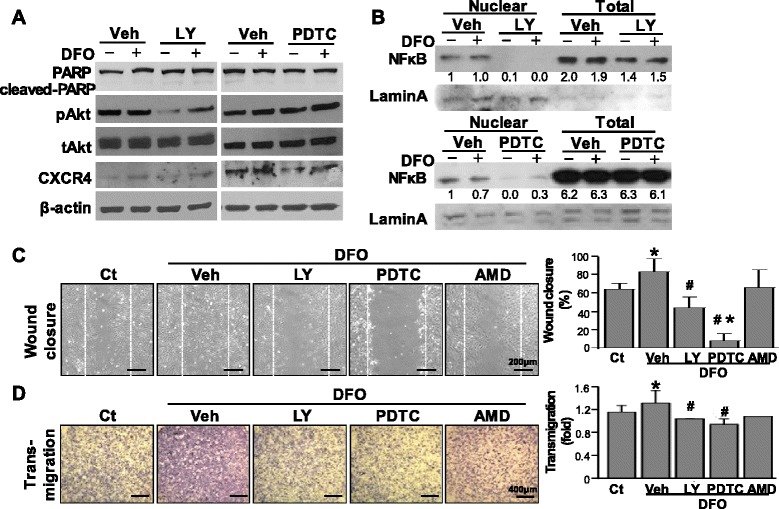
Inhibition of Akt and NFκB were administrated by applying LY294002 (*LY*) and PDTC, respectively. CXCR4 expression in eELCs was inhibited by both LY and PDTC (**a**). The nuclear translocation of NFκB was inhibited by PDTC treatment under hypoxia and was totally abolished when treating with LY in both normoxia and hypoxia conditions (**b**). The increase of wound closure in eELCs under hypoxia (DFO) was inhibited by LY and almost abolished by PDTC treatment (**c**). The increase of transmigration ability in hypoxic eELCs was also inhibited by either LY or PDTC inhibitor (**d**). *Scale bar*: (**c**) 200 μm; (**d**) 400 μm. *Significant from eELCs under regular culture condition (without DFO), *p* < 0.05; #significant from eELCs under hypoxia (DFO, 50 μM), *p* < 0.05. *AMD* AMD3100, *CXCR4* C-X-C chemokine receptor type 4, *DFO* desferrioxamine, *PARP* poly-ADP ribose polymerase, *PDTC* pyrrolidinedithiocarbamate, *Ct* control, *Veh* vehicle

To understand potential signaling for the enhancement of migration in eELCs, total proteins were isolated from MSCs and eELCs with or without DFO treatment. The eELCs showed higher CXCR4 protein expression than MSCs under both normoxia and hypoxia conditions (Fig. 4a). The potential intracellular pathways were assessed by observing the Akt, NFκB p65, and IκBα signals in MSCs and eELCs (Fig. 4b). In MSCs, phospho-Akt (pAkt) was increased under DFO treatment for 24 hr. The eELCs showed higher protein expression levels in pAkt, NFκB p65, and IκBα than MSCs. However, the application of DFO did not alter these protein expressions in eELCs. We further used LY294002 (LY) to inhibit PI3K/Akt signals and the PDTC to block the NFκB pathway with or without the presence of DFO (Fig. 5a). Both the LY and PDTC treatments did not increase cleaved PARP in eELCs under either the normoxia or hypoxia condition. CXCR4 protein expression was decreased with LY or PDTC treatment, showing that pAkt and NFκB signals might regulate CXCR4. The inhibition of PI3K/Akt or NFκB signals in eELCs inhibited the nuclear translocation of NFκB p65 (Fig. 5b). The promotion of the wound closure ability in eELCs under DFO treatment was inhibited by LY (significant deferent to hypoxia) and totally abolished by PDTC (significant different to both normoxia and hypoxia) (Fig. 5c). The transmigration of eELCs also diminished under both PDTC and LY treatments (Fig. 5d). We also tested the CXCR4 antagonist AMD3100 (AMD) in hypoxic eELCs, but did not observe significant inhibition of wound closure or transmigration ability. Taken together, these results indicated the involvement of Akt and NFκB signaling to regulate CXCR4 protein expression for the migration ability of eELCs.

## Discussion

Hypoxic–ischemic injury in stroke and myocardial infarction is well known with many stem cell treatments. Nowadays, combinations with different gene manipulations or drug preconditions in stem/progenitor cells are mostly used to study the therapeutic effect on in-vivo ischemic injury [40–42]. However, the concern of gene manipulation and its safety for transplanting cells are always raised in clinical trials. Hence, the current study prefers to induce MSCs toward the endothelial lineage via the culture medium that contains endothelial growth factors.

Ischemia, including hypoxia, serum deprivation, and glucose deprivation, is usually involved in injured tissues. HIF-1 is a critical transcription factor in the hypoxic environment. The HIF-1 α subunit (HIF-1α) has a very short half-life in a normoxic environment which can be degraded by oxygen-dependent prolyl hydroxylase domain enzymes through the ubiquitin–proteasome pathway [43]. DFO and cobalt chloride (CoCl_2_) can stabilize HIF-1 protein for mimic hypoxia conditions in vitro. Other in-vitro hypoxia models were generated by reducing the oxygen content from 10 to 1% [44, 45] or deprivation of serum to damage stem/progenitor cells [21]. However, the hypoxia model using serum deprivation induced MSCs to undergo apoptosis and might not represent a physiological condition because serum is present in our body composition [46]. In this study, we use DFO to mimic an in-vitro hypoxic condition which aligned with the data from our previous study [37]. Our data suggested that hypoxia created by DFO increased cell migration, especially in eELCs. We also incubated the MSCs and eELCs in a low-oxygen hypoxia chamber and obtained similar results (Additional file 2: Figure S2). The hypoxic microenvironments enhanced the 3D encapsulated MSCs to promote the outgrowth of tube-like structures in PEGylated fibrin and to secrete VEGF and MMP2 [47]. In current study, the MSC response under hypoxia was consistent with these published works.

CXCR4 is a seven-transmembrane G protein-coupled receptor that the binds to SDF-1. SDF-1, also known as CXCL12, is a small secreted chemokine protein belonging to the CXC chemokine family. Several signaling cascades are activated after SDF-1 and CXCR4 form ligand–receptor complexes. The PI3K/Akt/eNOS signal pathway is involved in the SDF-1-induced EPC migration and MSC survival [48, 49]. Our data showed that DFO promotes Akt phosphorylation in MSCs (Fig. 4b) and the higher expression of pAkt for cell migration in ELCs was blocked by Akt inhibitor (Fig. 5). NFκB is a critical transcription factor involved in biological responses which includes immune responses, cell survival, stress responses, and maturation of various cell types. Five subunits of NFκB (RelA (p65), RelB (p100), cRel, p50, and p52) generate different dimeric complexes, and control cellular function through canonical or noncanonical pathways [50]. IκB phosphorylates and inactivates the NFκB signal for inflammatory and survival gene transcriptions. Under hypoxia, HIF-1α was correlated with NFκB due to the inhibition of oxygen-dependent hydroxylases for activation of both the HIF-1 and NFκB pathways [16, 51]. Although our results showed that NFκB and IκB were not altered by DFO treatment in both MSCs and eELCs (Fig. 4b), the DFO-mediated cell migration was abolished by inhibiting the NFκB signaling using PDTC (Fig. 5). The results of decreasing the NFκB nuclear translation under both Akt and NFκB inhibitor treatments confirmed the essential role of Akt/NFκB signaling in eELC migration.

Besides the migration ability for therapeutic cell homing to the damaged tissue, the angiogenesis process is also very important for repairing injury. The reendothelialization capacity was improved by recruiting EPCs via CXCR4 [52] and PI3K/Akt [53] signaling. Overexpression of CXCR4 in MSCs facilitated the treatment of acute lung injury in rats [54]. The angiogenic function of EPCs also associated with PI3K/Akt when treating the conditioned medium isolated from multipotent stromal cells [15]. We demonstrated an increase of CXCR4 and pAkt protein expressions in eELCs (Fig. 4) which might benefit the angiogenesis in vivo. Shear stress can also recruit PI3K and induces NFκB translocation and transcriptional activity via the integrins/FAK/actin network [55, 56]. The importance of mechanical factors on MSC differentiation was summarized in a recent review [57]. LSS, a mechanical force on the straight part of vascular endothelium, has vasoprotective function, suppresses inflammatory response [58], and promotes differentiation and tube formation on EPCs [59]. However, the angiogenic potential and functions in early and late EPCs are divergent [60]. Early EPCs secrete plentiful cytokines, including VEGF, interleukin-8, hepatocyte growth factor, and granulocyte-colony stimulating factor [60, 61], while late ELCs have a better ability in proliferation and endothelial incorporation [60]. In the current study, eELCs showed characteristics similar to early EPCs, whereas LSS facilitated these cells toward late EPCs or mature ECs. Although LSS benefits endothelial maturation (Fig. 3), the decrease of transmigration ability after subjecting eELCs to LSS suggested that these endothelial therapeutic cells in a distinct differentiation phase may have a unique role in protecting the vascular structure.

## Conclusion

In this study, we demonstrated that MSCs can differentiate into endothelial lineage and promote migration ability in responding to a hypoxic microenvironment, which might benefit cell therapy and tissue regeneration. DFO is on the List of Essential Medicines as announced by the World Health Organization to demonstrate its safety and importance in medications, especially for treating acute iron poisoning in small children. eELC induction and then preconditioning to DFO can provide a novel and convenient-to-clinics therapeutic strategy to increase the function of MSCs. In summary, we conclude the eELCs derived from MSCs may serve as a better source for cell-based therapy regarding hypoxia resistance and migration ability.

## Additional files

Additional file 1: Figure S1. showing similar cell proliferation abilities were observed by BrdU (**A**) and Ki67 (**B**) staining in both MSCs and eELCs. (PDF 161 kb)
Additional file 2: Figure S2. showing the hypoxia incubator has similar gene expression patterns in both MSCs and eELCs as compared withto these observed in DFO treatments (**A**). Same induction effects of p-Akt in hypoxic MSCs and increase of CXCR4 in eELCs were confirmed when incubating the cells in a low oxygen incubator (**B**). (PDF 237 kb)
