# Supplementary figures and images for: Endothelial differentiation of bone marrow mesenchyme stem cells applicable to hypoxia and increased migration through Akt and NFκB signals
Source: Stem Cell Res Ther. 2017 Feb 7;8:29. doi: 10.1186/s13287-017-0470-0 (PMC5296962; doi:10.1186/s13287-017-0470-0)

Additional file 1: Figure S1

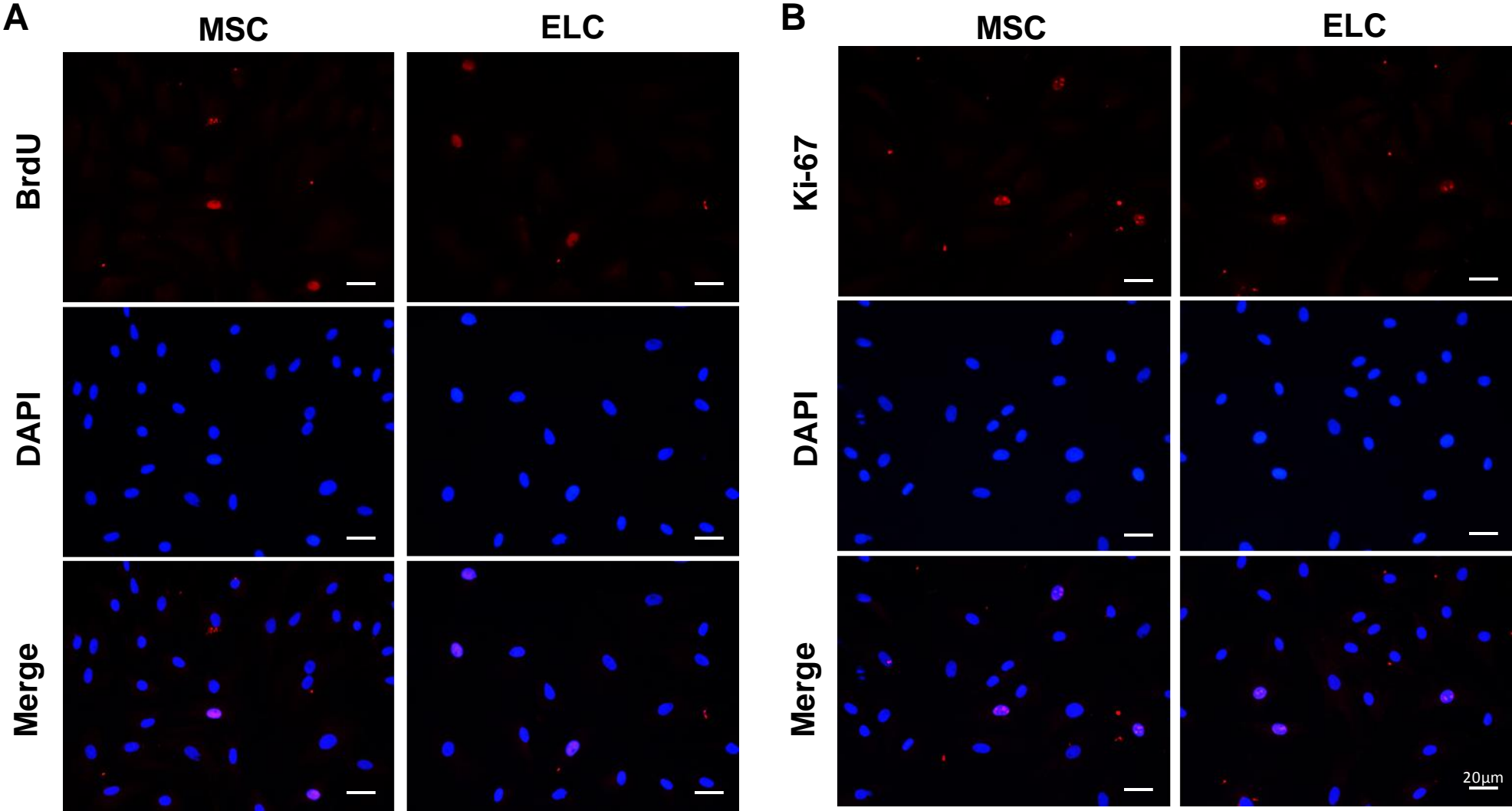

Supplement: Additional file 1: Figure S1. — showing similar cell proliferation abilities were observed by BrdU (A) and Ki67 (B) staining in both MSCs and eELCs. (PDF 161 kb) [file 13287_2017_470_MOESM1_ESM.pdf]

**A**

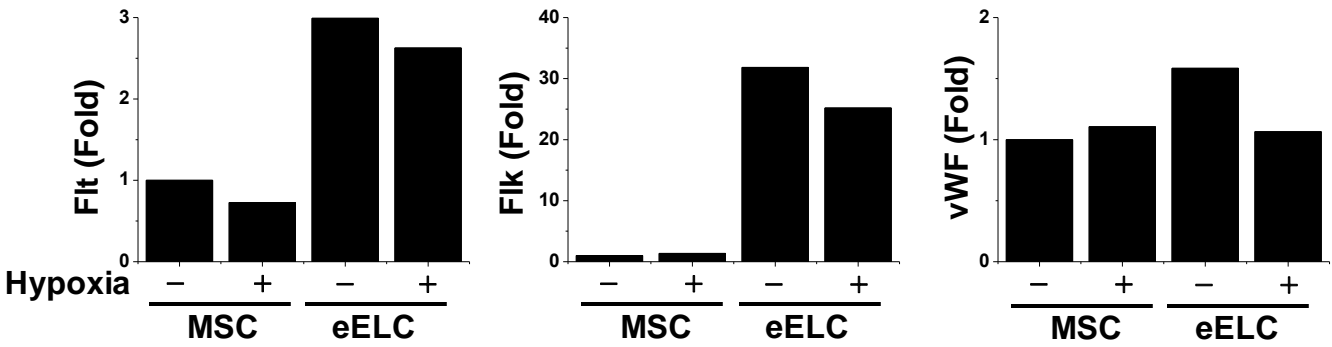

**B**

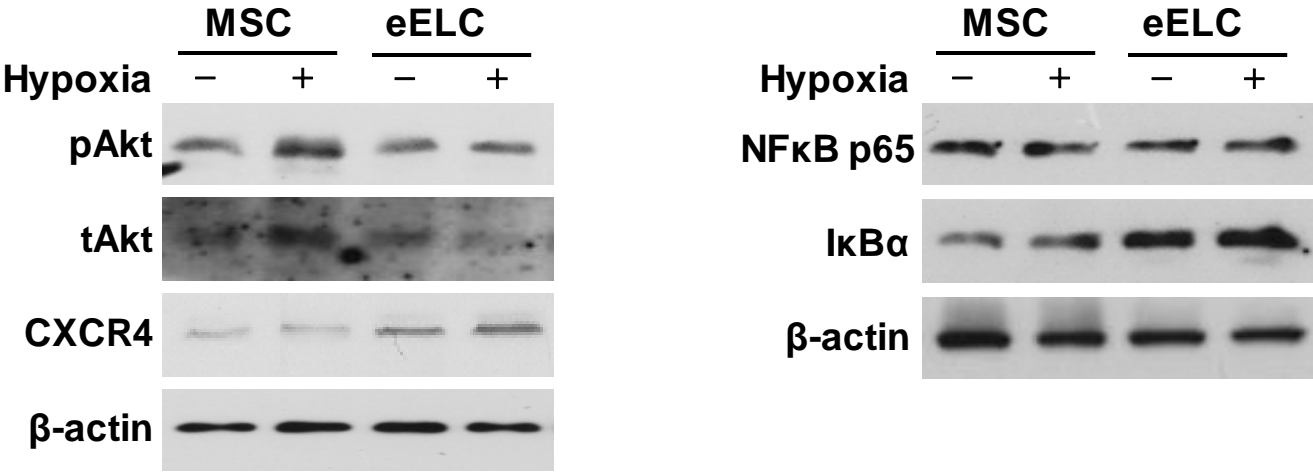

Supplement: Additional file 2: Figure S2. — showing the hypoxia incubator has similar gene expression patterns in both MSCs and eELCs as compared withto these observed in DFO treatments (A). Same induction effects of p-Akt in hypoxic MSCs and increase of CXCR4 in eELCs were confirmed when incubating the cells in a low oxygen incubator (B). (PDF 237 kb) [file 13287_2017_470_MOESM2_ESM.pdf]
